# Criteria and Guidelines for Returning to Running Following a Tibial Bone Stress Injury: A Scoping Review

**DOI:** 10.1007/s40279-024-02051-y

**Published:** 2024-08-14

**Authors:** Esther R. M. George, Kelly R. Sheerin, Duncan Reid

**Affiliations:** 1 Sports Performance Research Institute New Zealand (SPRINZ), Faculty of Health and Environmental Sciences, Auckland University of Technology, Auckland, New Zealand; 2 InForm Physio, Silverstream, New Zealand

## Abstract

Tibial bone stress injuries (BSIs) are common among long-distance runners. They have a high recurrence rate, and complexity emerges in the wider management and successful return to running. Following a tibial BSI, a critical component of complete rehabilitation is the successful return to running, and there is a lack of consistency or strong evidence to guide this process. The objectives of this review were to outline the criteria used in clinical decision-making prior to resuming running, and to establish evidence-based guidelines for the return to running process following a tibial BSI. Electronic databases including MEDLINE, CINAHL, Scopus, SPORTDiscus and AMED were searched for studies that stated criteria or provided guidelines on the objectives above. Fifty studies met the inclusion criteria and were included. Thirty-nine were reviews or clinical commentaries, three were retrospective cohort studies, two were randomised controlled trials, two were pilot studies, one was a prospective observational study, and three were case studies. Therefore, the recommendations that have been surmised are based on level IV evidence. Decisions on when an athlete should return to running should be shared between clinicians, coaches and the athlete. There are five important components to address prior to introducing running, which are: the resolution of bony tenderness, pain-free walking, evidence of radiological healing in high-risk BSIs, strength, functional and loading tests, and the identification of contributing factors. Effective return to running planning should address the athlete’s risk profile and manage the risk by balancing the athlete’s interests and reinjury prevention. An individualised graduated return to running programme should be initiated, often starting with walk-run intervals, progressing running distance ahead of speed and intensity, with symptom provocation a key consideration. Contributing factors to the initial injury should be addressed throughout the return to run process.

**Supplementary Information:**

The online version contains supplementary material available at 10.1007/s40279-024-02051-y.

## Key Points

**Table:** 

The decision on when to start the return to running process following a tibial bone stress injury (BSI) should be purposeful and based on defined criteria.
The return-to-run process following a tibial BSI needs to be individualised, and based on multiple factors including the severity of injury and experience of the runner. While widely cited, the 10% rule of graduated loading is not generalisable to all runners.
It is important to acknowledge and address mechanical loading issues, and other contributing factors including biomechanical, nutritional and hormonal factors, particularly in females.

## Introduction

Bone stress injuries (BSIs) predominantly occur in physically active individuals, and are fundamentally due to an inability of normal bone to withstand repetitive loading [1]. There is a growing consensus BSIs occur due to an accumulation of load-induced microdamage that exceeds bone remodelling [2]. BSI is a holistic term that encompasses low-grade stress reactions through to fractures of the bone [3].

Up to 95% of BSIs in athletes occur in the lower extremities, with the tibia the most common location [4–10]. Lower extremity BSIs are common among distance runners due to the repetitive loading of the sport, with an annual incidence of as high as 21.1% found in track and field athletes and more than one-third of female cross-country and long-distance runners experiencing lower extremity BSIs [11, 12]. A systematic review of BSI incidence in military and athletic populations reported an overall incidence of 9.7% in female athletes and 6.5% in male athletes [13]. Tenforde et al. [10] found that tibial BSIs were the most common overuse injury sustained among competitive high school runners (lifetime prevalence of 41% of females and 34% of males). Additionally, BSIs have one of the highest recurrence rates of all running-related injuries [8, 14, 15]. Prior BSI has been shown to increase the recurrence rate sixfold in females and sevenfold in males [14].

Low energy availability (LEA), or more specifically Relative Energy Deficiency in Sport (REDs), is another intrinsic risk factor [16]. REDs expands on the Female Athlete Triad and recognises that both females and males can be affected. REDs refers to the physiological and psychological consequences as a result of the mismatch between energy intake and energy expenditure [17]. LEA contributes to impaired bone health and risk of BSIs, and commonly affects runners [18–20]. Amenorrhea and low testosterone, indicating chronic energy conservation, were found in 37% and 40% of elite female and male distance runners, respectively, and resulted in 4.5-fold higher rates of bone injuries than in runners with normal menstruation or testosterone levels [21].

Tibial BSIs can be classified based on grade of injury as well as risk of location. Several MRI BSI grading scales have been proposed, mostly grading injuries from 1–4 [22–24]. For most classification systems, the first three grades are considered ‘stress reactions’, and when there is a visible fracture line, the injury is considered a ‘stress fracture’, and typically classified as grade 4 [22–25].

BSIs can also be clinically classified as ‘low-risk’ or ‘high-risk’ injuries by anatomic location, which will guide treatment [1, 26–28]. In the tibia, the most common location especially among runners is the posteromedial tibial shaft, which is considered a low-risk injury. These typically heal without major complications and gradual return to running can be initiated earlier [1, 27]. In contrast, high-risk injuries involving the anterior tibial cortex may require surgical fixation or prolonged non-weight bearing, have a higher complication risk, and will require a longer timeline for returning to running [26]. Differences in grade of BSI or risk of location will result in differing recovery times to the point of starting the return to running process.

Tibial BSIs can result in disruption to activities of daily living, lost training time, considerable financial burden in elite athletes, and substantial reductions in cardiovascular and muscular function [8, 29, 30]. Therefore, it is important that management of these injuries is optimised. Following a tibial BSI, a critical component of complete rehabilitation is the successful return to running. However, there is a lack of evidence-based guidelines regarding when the athletes should begin this process and a lack of consistency or strong evidence to guide the process of returning to running. Tibial BSIs are a unique injury, which is one of the reasons why prevention and returning athletes to running following injury is so complicated. While existing reviews have explored the general concepts of BSI management [9, 13, 31–60], no criteria or guidelines have been established regarding when it is safe for athletes to return to running following a tibial BSI. The high recurrence rate among both female and male athletes indicates this process needs to be improved. Therefore it would be highly valuable to establish evidence-based clinical guidelines for the process of returning athletes to running and reduce the recurrence rates of tibial BSIs in runners.

The specific aims of this scoping review are:

1. To outline the criteria used in clinical decision-making prior to resuming running following a tibial bone stress injury.
2. To establish evidence-based guidelines to support clinicians in the return to running process following a tibial bone stress injury.

## Methods

The methodological framework proposed by Arksey and O’Malley [61] and the JBI Evidence Synthesis [62] were followed for the design and reporting of this scoping review: step 1, identify the research question; step 2, identify relevant studies; step 3, study selection; step 4, charting the data; and step 5, collating, summarising and reporting the results.

Initial literature searches revealed few papers specific to returning to running following tibial BSIs, and as such the search scope was widened to include lower extremity BSIs, and any return to running-based activities. Studies included sources of information as recommended in the manual ‘Methodology for JBI Scoping Reviews’ [62] that provided guidelines for the process of returning to running-related activities or stated criteria prior to introducing running-related loads. Only full-text studies published in English were included. Keywords and constructs (i.e., MeSH, Boolean phrases) used to execute each search were developed from a preliminary search (Table 1), and the full search strategies for all databases can be found in the Online Supplementary Material (OSM) File 1. The reference lists of included studies and the reference lists of key reviews were also screened, and a forward citation-tracking Google Scholar was conducted to identify any potentially relevant studies that may have been missed in the database search [50]. Studies were included if they outlined specific criteria prior to the introduction of running-related loads, or provided guidance on the process of returning to running-related activities, following a tibial or lower extremity BSI (Table 2).

**Table 1:** Scoping review search terms

Search 1	Search 2
"Bon* Stress Injur*" OR "Stress fracture*" OR "Stress reaction*"	"Bon* Stress Injur*" OR "Stress fracture*" OR "Stress reaction*"
"lower extremit*" OR "lower limb*" OR leg* OR knee OR tibia*	"lower extremit*" OR "lower limb*" OR leg* OR knee OR tibia*
(return*) n3 (sport* OR play OR training OR activit*)	(Run*)

**Table 2:** Inclusion and exclusion criteria

Inclusion criteria	Exclusion criteria
Human participants	Animal models or cadavers
Tibial or general lower extremity bone stress injuries	Upper extremity or spinal bone stress injuries, or specific lower extremity bone stress injuries other than the tibia
Describes the activities, process, or criteria prior to beginning the process of returning or running-related activities	No description of the return to running-related activities, process, or criteria prior to beginning running-related activities
Specific detail of bone stress injury management	No specific mention of bone stress injury management

**Fig. 1:**
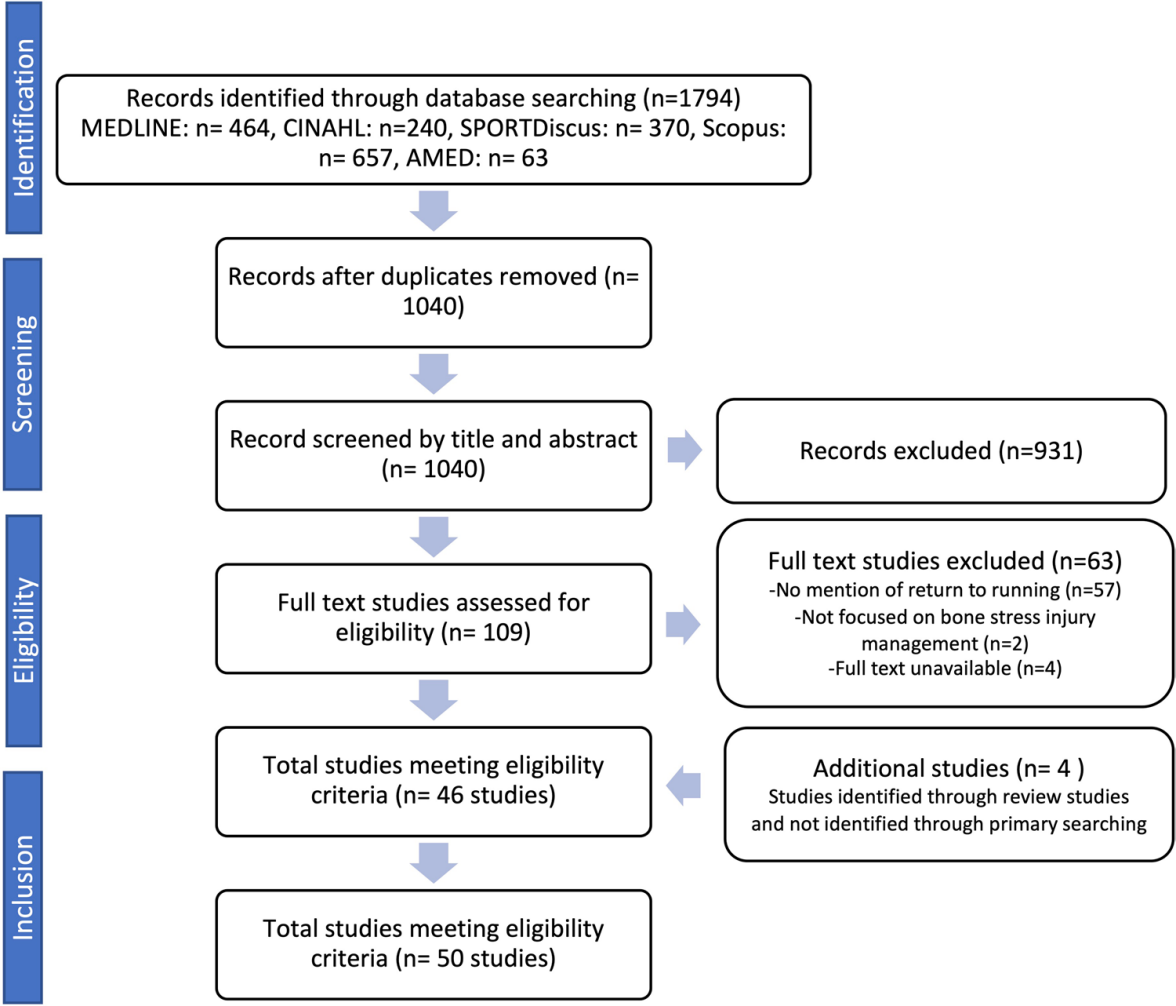
Preferred Reporting Items for Systematic reviews and Meta-Analyses (PRISMA) flow diagram

The lead author (EG) screened titles and abstracts, and EG and KS then independently screened the full-text articles to determine the final study selection (Fig. 1). Any discrepancies were resolved during a consensus meeting. A third reviewer (DR) was available if needed, but was not required. Data were extracted into a spreadsheet by EG, and independently verified by KS. Disagreements were resolved via consensus or discussion with DR. An inductive thematic analysis was used to identify patterns, summarise consistent findings across studies, and generate common themes [63]. Regular meetings were held to discuss and agree on emerging themes and interpretations.

## Results

The initial search identified 1040 studies, of which 50 studies met the inclusion criteria (Fig. 1). Of these, 39 were reviews or clinical commentaries, three were retrospective cohort studies, two were randomised controlled trials, two were pilot studies, one was a prospective observational study, and three were case studies. The variation in the quality of these studies further justified that a scoping review was appropriate rather than a systematic review. Of the 50 studies, 48 provided criteria prior to introducing running-related loads, and four themes of criteria were identified. All studies provided guidance on the return-to-run process, and three themes were identified.

### Return-to-Run Criteria

#### Symptom Resolution

All studies that identified criteria indicated that the athlete must first be pain-free, and their symptoms resolved with ambulation and activities of daily living for a period ranging from 2–28 days prior to the introduction of running-related loads [2, 5, 22, 26, 27, 33, 34, 36, 38, 41, 43, 45, 49, 52–55, 64–88] (Table 3). Thirty percent of reviewed studies recommended a certain walking distance or duration ranging from 1 mile to 45 min as presented in Table 3 [2, 33, 54, 65, 66, 68, 70–72, 76, 81, 85, 89, 90]. Two reviewed studies recommended completing this walking distance three times [72, 85]; however, the number of repetitions was not specified in any other studies.

**Table 3:** Return-to-run criteria themes

Recommendation	Detail	References
Resolution of bony local tenderness		[2, 34, 41, 43, 55, 68–72, 74, 77–83, 86]
	For 1 week	[36, 52, 73]
	Not required	[5, 54]
Pain-free with ambulation and activities of daily living		[26, 27, 33, 38, 55, 67–72, 75–81, 84, 88]
	2-day pain-free duration	[66]
	3- to 5-day pain-free duration	[2, 22, 54, 64, 65, 91]
	10- to 14-day pain-free duration	[5, 34, 36, 41, 43, 45, 49, 52, 53, 73, 74, 85, 86]
	3–4 weeks	[87]
Pain-free walking	30 min	[2, 65, 85]
	45 min	[89]
	2 × 20 min	[90] (pain less than 3 on visual analogue scale)
	1 mile	[54, 66]
	1.5 miles	[72]
	3× weekly	[72, 85]
Evidence of radiological healing	Not necessary for low-risk locations (posteromedial tibial)	[5, 27, 33, 34, 36, 45, 52, 68, 70, 72, 77, 82, 84, 89]
	Only necessary in high-risk locations (anterior tibial cortex)	[26, 36, 45, 52, 68, 70, 72, 77, 79, 84, 88]
	Necessary	[53, 80, 86, 87]
	Referral for imaging if return of symptoms	[43]
Functional movements assessed	Yes	[2, 22, 38, 52, 73, 74, 76, 77, 86, 87, 89, 91]
	Hop	[36, 54, 65, 66]
	Single leg squat	[2]
Lower extremity strength assessed	Yes	[2, 22, 53, 73, 77, 80, 89, 92]
	75–85% strength	[41]
Contributing factors	Nutritional factors	[2, 16, 26, 27, 33, 34, 36, 38, 43, 45, 49, 52, 53, 55, 64, 65, 68–70, 72–74, 76–80, 82, 84, 86, 87, 93]
	Menstrual health/Relative Energy Deficiency in Sport	[2, 22, 26, 27, 33, 34, 36, 38, 45, 49, 52, 53, 55, 64, 65, 67–70, 72–74, 76–80, 82, 84, 86, 89, 93]
	Footwear	[2, 5, 26, 27, 33, 34, 38, 43, 45, 49, 53, 55, 64, 65, 68–70, 73, 74, 76, 77, 79–81, 84–87, 93]
	Training errors	[2, 5, 22, 26, 27, 33, 34, 38, 43, 45, 49, 53, 55, 64, 65, 68–70, 72–74, 76–78, 80, 81, 84, 87, 89, 91–93]
	Psychological factors	[16, 73, 79]
	Biomechanical factors	[2, 5, 16, 26, 27, 33, 34, 36, 38, 41, 43, 45, 52, 53, 55, 64, 65, 67–81, 83–87, 89, 91–94]

Forty percent of reviewed studies stated that bony tenderness should have resolved before introducing running-related loads [2, 34, 41, 43, 55, 68–72, 74, 77–83, 86]. Three studies, which were either clinical commentaries or reviews, specified this should be for at least 1 week [36, 52, 73], while a randomised controlled trial (RCT) and case series presented contrasting recommendations, indicating that persistent localised tenderness did not influence initiation or completion of the running progression with participants [5, 54].

#### Radiology

Differing recommendations were provided regarding the requirement for radiologic healing prior to introducing running-related loads (Table 3). Ninety-one percent of studies either did not state imaging as a criterion prior to introducing running, or specified that evidence of radiological healing was not required prior to introducing running-related loads when dealing with low-risk BSI locations, due to the lack of consistency between clinical and radiological healing and the limited sensitivity of radiographs [5, 27, 33, 34, 68, 70, 72, 82, 84, 89]. In the case of a high-risk BSI, such as anterior tibial cortex BSI, 23% of studies specified imaging as important to confirm healing and prevent complications such as progression to full fracture before the athlete returns to running [26, 36, 45, 52, 68, 70, 72, 77, 79, 84, 88]. The studies providing recommendations on the inclusion of imaging were all either clinical commentaries, reviews or case studies.

#### Strength, Functional and Loading Tests

Thirty-three percent of studies indicated lower extremity functional movements should be assessed prior to introducing running-related loads [2, 22, 36, 38, 52, 54, 65, 66, 73, 74, 76, 77, 86, 87, 89, 91] (Table 3). Only a small number of specific movements were recommended, with a single leg hop (SLH) test to indicate preparedness for return to running-related activities reported by only 8% of studies [35, 54, 65, 66]. An RCT by Swenson et al. [54] reported that the SLH test was strongly correlated with functional progression, and was the most sensitive test for predicting the return to unrestricted pain-free activity [54]. The remainder of the studies were clinical commentaries or reviews. Nine studies indicated lower extremity strength should be assessed and addressed prior to introducing running-related loads [2, 22, 41, 53, 73, 77, 80, 89, 92]. Harmon et al. [41] specified 75–80% of lower extremity strength of the injured compared to the non-injured side should be achieved prior to introducing running-related loads.

#### Addressing Contributing Factors

All studies that stated return to running criteria agreed that a key component of management is to identify and address contributing factors involved in the initial development of the BSI [2, 5, 16, 22, 26, 27, 33, 34, 36, 38, 41, 43, 45, 49, 52, 53, 55, 64, 65, 67–89, 91–93]. These factors will be individual to the athlete, but important factors identified included menstrual health, nutritional deficiencies, energy availability, running biomechanics, muscle strength and flexibility deficits, mechanical loading issues (commonly referred to as ‘training errors’) and footwear to prevent the risk of recurrence (Table 3). Sixty percent of studies identified the importance of assessing REDs risk and menstrual health when treating a female athlete [2, 26, 27, 33, 34, 36, 38, 45, 49, 52, 53, 55, 64, 65, 67–70, 72–74, 76–80, 84, 86, 93]. It is indicated that these contributing factors should be addressed prior to a return to full training.

### Return-to-Running Process

#### Graduated Running Progression

The athlete’s goals and previous running level should guide the return to running process [33, 34, 41, 68, 73, 74, 81, 85]. A walk-run progression, gradually substituting walking with increasing time increments of running, was recommended by 26% of reviewed studies that provided guidance on return to running [2, 33, 54, 64, 66, 68, 70–72, 77, 85, 89, 90]. Two RCTs and one pilot study presented walk-run programmes and the rest of these recommendations were from review studies or clinical commentaries. The length of running increments ranged from 100 to 5 min, and the progression of these increments ranged from 100 to 5 min (see Table 3). The initial speed of these running increments will be dependent on the pre-injury level of the runner, but 46% of studies recommended beginning at a slower pace, with specific recommendations presented in Table 3 [2, 33, 36, 38, 43, 45, 52, 54, 64–66, 68, 70–73, 77, 83, 85, 89, 90, 92].

Of the studies that stated a frequency of initial runs, 42% recommended starting with alternate days [2, 5, 22, 36, 45, 52, 53, 55, 64, 65, 72–74, 76, 81, 83, 85, 87, 90–92], with several studies specifying this should be maintained for times ranging from 2 [36, 73, 74] to 4 weeks [2, 64] (Table 3). Other studies indicated the athlete could start at an increased frequency depending on symptoms [33, 54, 66, 68, 70, 71, 89].

Progressively increasing running distance by 10% per week, commonly referred to as ‘the 10% rule’, is a common recommendation to prevent injury during normal training [72, 80], but is also widely cited as a method of returning to running following a lower extremity BSI [34, 38, 41, 43, 45, 55, 56, 65, 77, 78, 81, 83, 86, 93] (Table 3). A small number of these clinical commentaries or reviews highlighted that this approach is not generalisable, and individual runners may tolerate different rates of progression [2, 41, 85], but provided no more specific guidance. Pain or symptom provocation were the main indicators used in the reviewed studies to guide the progression through the return-to-running process following a tibial BSI [2, 5, 16, 22, 33, 36, 38, 41, 43, 45, 53, 54, 64–66, 68, 70–72, 74–78, 80–82, 84–93], with several studies specifying the importance of being pain-free, both during and following activity [2, 45, 65, 78]. If symptoms were provoked at the injury site whilst running, it was recommended that athletes rest until symptoms resolved, and then resume at a lower level [2, 16, 22, 33, 41, 64, 68, 70–72, 74, 76, 77, 86, 89–91, 93].

It is widely cited that distance should be progressed prior to increasing speed when returning to running following a tibial or lower extremity BSI [2, 16, 33, 34, 45, 54, 64, 65, 68, 70, 71, 73, 74, 81, 85, 89, 90] (Table 3), with 11 studies suggesting a specific running distance that should be achieved before speed changes, ranging from 1 mile to 45 min [2, 33, 54, 64, 66, 68, 70, 71, 74, 89, 90]. Similar to the progression of distance, five studies suggested ‘the 10% rule’ as a guideline to progress running speed [2, 38, 43, 77, 86]. Other recommendations regarding the progression of speed are presented in Table 3.

#### Running Surface

While a number of studies provided specific recommendations to initiate running on either a treadmill [33, 34, 68, 70, 71, 89, 93] or a running track [54, 66, 90], there were conflicting recommendations regarding return to running and surfaces (Table 3). Other recommendations included starting on a level surface or limiting hills during recovery [2, 38, 43, 52, 67, 73, 77], and avoiding hard [2, 36, 52, 55, 64, 73, 74, 76, 79, 80, 82, 84, 85, 93] or uneven [2, 38, 67, 76, 77, 80, 82] surfaces. Some studies suggested avoiding multiple terrains during the initial recovery [2, 43, 64, 85], while others recommended varying terrain once back to normal training [16, 38, 64, 67, 89]. Two RCTs and one pilot study recommended introducing running-related loads on the running track. The remainder of studies providing recommendations were review studies or clinical commentaries.

#### Biomechanics and Strength Training

An important component of the return-to-running process recognised by 62% of reviewed studies was to address lower extremity biomechanical abnormalities thought to contribute to the initial injury [2, 16, 22, 33, 34, 36, 38, 41, 43, 45, 49, 52, 53, 64, 65, 67–70, 72–78, 81, 84, 87, 89, 91].

Furthermore, muscle strengthening was identified as important by 74% of studies to correct muscle imbalances and improve biomechanics following a tibial BSI [2, 5, 16, 22, 33, 34, 38, 41, 43, 45, 52, 53, 55, 64, 65, 67–73, 76–81, 83–87, 89, 91–93]. Strengthening of local muscles including the calf and tibialis anterior [2, 5, 33, 38, 43, 45, 52, 55, 64, 65, 71, 77, 85, 93], as well as proximal strength, including the core and pelvic muscles [2, 43, 45, 64, 65, 77, 80, 81], were recommended (Table 3). Eleven studies acknowledged the importance of progressing to plyometric strengthening and including running drills in this process [33, 41, 45, 64, 65, 68, 70, 81, 85, 89]. It was suggested that these recommendations were more specific once athletes could sprint [33, 68, 70, 89] or squat one and a half times their body weight [85]. Lastly, 28% of studies identified addressing muscle flexibility as an important component [5, 33, 34, 38, 43, 45, 53, 55, 64, 67, 77, 80, 86, 94], in particular calf and hamstring flexibility in the case of a tibial BSI. The majority of the studies providing recommendations on biomechanics and muscle strengthening were clinical commentaries, case studies or reviews (Table 4).

**Table 4:** Return-to-run process themes

Component of process	Recommendation	Detail	References
Introduction of running load (time/distance)	Walk-run		[2, 33, 54, 64, 66, 68, 70–72, 77, 81, 85, 89, 90]
		Start with 30-s running increments	[81]
		Start with 100-m running increments	[54, 85, 90]
		Start with 1-min running intervals	[2, 64, 72]
		Start with 400-m running increments	[66]
		Start with 5-min running increments	[33, 68, 70, 71, 74, 89]
		Progress running increments by 1–2 min	[2, 64, 72]
		Progress running increments by 100–400 m	[54, 66]
		Progress running increments by 5 min	[33, 68, 70, 71, 74, 89]
		Progress total distance but keep the same running increments length	[85, 90]
	Gradual progression		[5, 16, 26, 27, 36, 43, 49, 52, 67, 69, 73, 75, 76, 79, 80, 84, 92, 93]
	Alternate days		[5, 22, 45, 52, 53, 55, 65, 72, 76, 81, 83, 85, 87, 90–92]
		For first 4 weeks	[2, 64]
		For first 2 weeks	[36, 73, 74]
	Rest days included	During progression	[67, 77, 84]
		During normal training	[26, 27]
	Daily depending on symptoms		[33, 54, 66, 68, 70, 71, 89]
	10% progression		[34, 38, 41, 43, 45, 55, 56, 65, 77, 78, 81, 83, 86, 93]
		As part of usual training/for injury prevention	[72, 80]
		Acknowledges lack of evidence	[56, 78, 81]
		Not generalisable, runners may tolerate different rates of progression	[2, 41, 85]
	15–20% progression Progression guided by pain		[85]
			[5, 36, 38, 43, 52, 53, 55, 71, 74, 75, 80–85, 87, 88, 92]
		If pain rest and resume at a lower level	[2, 16, 22, 33, 41, 54, 64–66, 68, 70, 72, 76, 77, 86, 89, 91, 93]
		If pain less than 3/10 at rest and resume at lower level	[90]
		Specify the athlete should be pain-free during and following	[2, 45, 65, 78]
	Progression guided by goals and previous running level		[22, 26, 27, 33, 34, 41, 49, 68, 70, 73, 74, 81, 82, 85, 86, 89, 91]
	Progression guided by whether location is low-risk (posteromedial) or high-risk (anterior tibial cortex)		[2, 26, 27, 33, 34, 36, 41, 45, 52, 53, 65, 69, 71–74, 76–79, 82, 84, 93]
	Progression guided by grade/severity of injury		[2, 16, 22, 33, 34, 43, 52, 64, 65, 70, 73, 77, 79, 81, 82, 84, 86, 90, 91]
Running speed	Start 30–50% usual pace		[2, 36, 38, 43, 45, 52, 65, 73, 77]
	Start at a slower pace		[16, 33, 54, 66, 68, 70–72, 83, 85, 89, 90, 92]
	Pace reduced by 1 m/s		[64]
Progression of speed	Progress from jogging to running		[41, 69]
	Progress from walk-jog to jog-run		[54, 90]
	Increase intensity by 10% weekly		[2, 38, 43, 77, 86]
	Progress to half pace strides then gradually progress to full pace striding		[33, 68, 70, 89]
	Increase distance prior to speed		[2, 16, 33, 34, 45, 54, 64, 65, 68, 70, 71, 73, 74, 81, 85, 89, 90]
Criteria prior to speed changes	45 min		[33, 68, 70, 71, 74, 89]
	40 min		[64]
	6 track laps walk/6 laps jogging (1.5 miles total)		[90]
	30 min		[2]
	1 mile		[54, 66]
	Temporarily reduce running volume when increasing speed		[65, 90]
	Hold distance steady when increasing speed		[2, 33, 54, 68, 70, 89]
Running surface	Start on level asphalt		[67]
	Start on running track		[54, 66, 90]
	Track may increase strain		[73]
	Start on treadmill		[33, 34, 68, 70, 71, 89, 93]
	Start on level surface		[38, 52, 67, 77]
	Start on moderate firmness surface		[38]
	Hard surfaces risk factor/ avoid		[2, 36, 52, 55, 64, 73, 74, 76, 79, 80, 82, 84, 85, 93]
	Start on softer surfaces initially		[74]
	Hills can increase strain/risk		[2, 38, 43, 73]
	Irregular/uneven/soft surfaces can increase strain/risk		[2, 38, 67, 76, 77, 80, 82]
	Limit multiple terrains initially		[2, 43, 64, 85]
	Vary terrain once back to normal training		[16, 38, 64, 67, 89]
Biomechanics	Address lower extremity biomechanics		[16, 22, 26, 33, 34, 38, 53, 55, 67, 68, 70, 79, 81, 84, 87, 89, 91]
	Gait retraining		[2, 36, 41, 43, 45, 64, 65, 67, 74, 76, 77, 79, 81, 91]
		Reduce stride length/ increase cadence	[2, 64, 65, 77]
		To reduce vertical loading rates	[2, 45]
		Modify initial foot contact	[2]
	Risk factors	Excessively supinated or pronated feet	[5, 33, 34, 36, 52, 68, 70, 73, 75, 76]
		Reduced dorsiflexion range	[34, 73]
		Increased peak hip adduction angle	[36, 43, 52, 74, 78]
		Increased rear foot eversion angle	[34, 36, 43, 52, 74, 78]
		Increased vertical loading rates	[36, 45, 52, 74]
		Increased rearfoot striking	[36, 45]
	Orthotics		[41, 65, 71, 90, 95]
Strengthening	Include strengthening		[2, 5, 16, 22, 33, 34, 38, 41, 43, 53, 55, 65, 67–73, 76, 78–81, 83, 84, 86, 88, 89, 91–93]
		Calf strength	[2, 5, 33, 43, 45, 52, 55, 64, 65, 71, 77, 85, 93]
		Hip strength	[2, 45, 64, 65]
		Dorsiflexor/ intrinsic foot muscle strengthening	[2, 38]
		Core strengthening	[2, 43, 45, 80, 88]
	Include balance		[43, 85]
	Plyometrics/running drills		[33, 41, 45, 64, 65, 68, 70, 81, 85, 88, 89]
		Include 3 × weekly	[65]
		Introduce once athlete can squat 1.5 times body weight	[85]
		Introduce once able to fully sprint	[33, 68, 70, 89]
Flexibility	Include flexibility		[5, 38, 43, 55, 64, 67, 77, 80, 86, 94]
		Calf stretching	[33, 34, 38, 45, 55, 64, 67]
		Hamstring stretching	[64, 94]

## Discussion

The main objectives of this scoping review were to summarise and make recommendations regarding firstly the return to running criteria currently used to safely return athletes to running, and secondly the guidelines for the process of returning athletes to running following a tibial BSI. With regard to the running criteria, five important components have been identified to address prior to returning athletes to running. These include the resolution of local bony tenderness, pain-free walking, evidence of radiological healing only in the case of a high-risk BSI, assessment of strength, functional and loading movements, and identification of contributing factors. There are then four important considerations in the return-to-running process. These include walk-run progression, progression of running load, running surface, and addressing biomechanical and strength factors.

### Return-to-Run Criteria

#### Resolution of Localised Tibial Tenderness

Tibial tenderness should be assessed by a medical professional, and then monitored by the athlete during daily activities, ambulation and rehabilitation [2, 34, 43, 68–70, 72, 74, 77, 86]. Localised tibial tenderness has been found to correlate with more involved marrow and cortical abnormalities findings on MRI [23], and therefore once bony tenderness has resolved a significant degree of healing should have occurred. There was no consensus between scoping review studies on whether complete resolution of bony tenderness is required. A number of reviews have suggested that resolution of bony tenderness is required prior to introducing running-related loads [2, 34, 36, 41, 43, 52, 55, 68–74, 78–83, 86]; however, there is a lack of scientific evidence to support these statements. Conversely, an RCT and a large case series reported that persistent localised tibial tenderness did not influence initiation or successful completion of the functional progression among their participants [5, 54]. Those who began the return-to-run process with bony tenderness still successfully completed the functional progressions [54]. Therefore, waiting for complete resolution of bony tenderness may unnecessarily prolong the return to running process following a low-risk tibial BSI. Following a low-risk BSI, a logical approach may be to assess bony tenderness and monitor for any increases throughout the process of increasing running load. Ensuring complete resolution of bony tenderness prior to returning to running is, however, an important criterion following a high-risk tibial BSI, considering the major risk of complications such as non-union.

#### Pain-Free Walking

The second step is to ensure athletes progress their walking tolerance prior to initiating running. There is consensus from all studies that the athlete should be pain-free with walking. This is a logical criterion, as bone pain generally indicates mechanical or chemical irritation and overload of the bone [2, 23, 37, 96]. Walking between 1 mile and 45 min has been suggested by a number of studies [2, 33, 54, 64, 66, 68, 70, 71, 74, 89, 90]; however, there is a lack of evidence to support this, and minimal guidelines have been provided on the frequency of walks. A similar criterion of increasing walking to 60–90 min daily for 3 weeks has been suggested following a sacral stress fracture in a female runner [97]. Tibial stress has been shown to significantly increase during running compared with walking, more so in females, highlighting the need to gradually expose bone to load to ensure bone adaptation and prepare for running-related loads and re-conditioning of other structures, such as muscles, tendons, and other connective tissues, especially if there has been a prolonged period of deloading [98]. Clinically increasing pain-free walking tolerance makes sense to ensure positive bone adaption; however, further evidence and clarification on this point is required and specific walking distances should be individualised based on the runner.

#### Evidence of Radiological Healing

The results of this scoping review indicate that evidence of radiological healing is not required except in the case of a high-risk BSI, such as one involving the anterior tibial cortex. It is well established that early presentation and low-grade BSIs are often missed on plain radiographs, and findings on imaging often lag behind clinical healing [6, 24, 44, 99–101]. Wright et al. [101] carried out a systematic review to determine the diagnostic accuracy of imaging modalities used to diagnose lower extremity stress fractures. The findings indicated that radiographs are limited by low sensitivity, particularly in the early stages of stress fracture, and in some cases may not reveal an existing stress fracture at any time. Therefore clinical, as opposed to radiological, signs of healing should guide the decision to introduce running-related loads in low-risk BSIs, such as posteromedial tibial BSIs.

However, in the case of an anterior tibial cortex BSI, imaging to confirm complete healing prior to returning to running is important. This is in line with other BSI management recommendations discussing low-risk and high-risk BSIs, as there is an elevated risk of complications at high-risk locations including fracture progression, refracture, delayed union and non-union [6, 28].

#### Assessment of Lower Extremity Strength and Functional Pain Provocation Loading Tests

The initial phase prior to returning to running can be utilised to strengthen local and proximal muscles. Only a relatively small number of studies specified assessing lower extremity strength prior to introducing running-related loads, with one study recommending achieving 75–80% lower extremity strength symmetry [41]. However, almost all reviewed studies recommended addressing biomechanical abnormalities and muscle imbalances thought to contribute to the initial injury. This is likely an important component as reduced muscle mass and strength have been identified as risk factors for tibial BSIs [11, 12, 95, 102, 103]. However, further research is required to identify specific strength criteria prior to returning to running following a tibial BSI.

Functional tests replicating some of the physical requirements of running may determine whether the athlete is ready to return to running. Although only one-third of studies indicated functional movements should be assessed, almost all reviewed studies recommended addressing biomechanical abnormalities and muscle imbalances thought to contribute to the initial injury. The SLH test replicates the loading and unloading components of running, and could assess the capacity of the tibia to withstand stress and readiness to return to running in conjunction with other components of the clinical examination [104, 105]. The SLH test is cited as a highly sensitive test for predicting the return to unrestricted pain-free activity, and is strongly correlated with functional progression following a tibial BSI in both female and male runners [54]. Similarly the SLH test has been used following femoral and sacral stress fractures to guide progression through the rehabilitation process [42, 106].

The 2016 Return to Sport Consensus Statement recommends that a battery of tests are used to mimic the demands of the sport when making a decision regarding return to sport [31]. Therefore based on the scoping review findings, along with other lower extremity BSI case studies and the Return to Sport Consensus Statement recommendations, clinicians could consider a battery of strength, functional and loading tests prior to introducing running-related loads. Assessments could include the SLH test, lower extremity strength evaluated by isometric assessments, and lower extremity biomechanical assessments. However, further research on functional tests required prior to returning to running is needed in athletes following a tibial BSI before clear guidelines can be provided.

#### Identification of Contributing Factors

The need to identify and address contributing factors such as menstrual health, REDs, nutritional factors, biomechanical factors, mechanical loading issues and footwear was acknowledged by reviewed studies. It is beyond the scope of this review to systematically review these risk factors, but these recommendations are in line with the intrinsic and extrinsic risk factors identified by Abbott et al. [8], as well as other reviews in this area [103, 107, 108].

Fundamentally all overuse injuries in runners are linked to mechanical loading issues [2, 73, 109, 110]. As such it is important that these are not repeated during the return to running process, or when full training eventually resumes. Alongside mechanical loading issues, it is particularly important that athletes are screened for REDs to optimise return-to-run outcomes, and to prevent long-term health consequences. REDs has a well-established effect on bone health and menstrual function in female athletes [20]. REDs and LEA should be screened for using the Low Energy Availability in Females Questionnaire (LEAF-Q), which has been validated in female athletes to classify energy availability [111]. Two risk-assessment tools have been introduced by the Triad Coalition and the International Olympic Committee (IOC) [17, 112, 113] to aid the clinical decision-making process, including regarding returning to sport, based on the level of risk. The Female Athlete Triad Coalition cumulative risk assessment tool identifies an athlete as of low, moderate or high risk for developing the Triad or REDs, and screens for factors that have been established in the literature to contribute to low bone mineral density and increased risk of BSIs [112]. This risk-scoring system has been shown to be predictive of BSIs, with female athletes classified as moderate or high risk having a 2.6- and 3.8-fold increased risk for BSI compared with athletes classified in the low-risk category [114]. The IOC REDs clinical assessment tool classifies athletes into low-, moderate- and high-risk categories depending on subjective and objective examination, particularly regarding the presence and causes of LEA, and aids return to sport decision-making [17]. It has not yet been validated for clearance to return to sport, and additional research is required to determine the tool’s predictive ability [115]. Therefore at this stage, the Female Athlete Triad Coalition cumulative risk assessment tool should be used in return-to-sport decision-making. However, future research would be beneficial to provide one updated and improved risk assessment scoring tool that can be utilised in both males and females to aid return to sport decision-making.

The many contributing factors associated with tibial BSIs highlight the importance of a multi-disciplinary approach to the return-to-running process including the involvement of registered dietitians, physiotherapy guidance, podiatry assessment, physiological assessment by a trained sports medicine physician, and possible referral for mental health counselling where applicable.

### Return-to-Run Process

#### Consideration 1: Walk-Run Progression

The initial introduction of running-related loads following a tibial BSI should be achieved through the progressive application of load to promote tissue adaption, whilst preventing injury reoccurrence. Typically, this is achieved via a walk-run progression on alternate days, gradually substituting walking with increasing time increments of running at a slower pace [2, 64, 90]. Several studies [33, 68, 70, 71, 89] all provide the same walk-run progression with no further evidence added since the initial proposition of this idea in the study by Brukner et al. in 1997 [68]. The specific length of running increments varied considerably among reviewed studies, however, starting with 30- to 60-s increments, and progressing by 1–2 min, or equivalent distance was suggested by half of the studies. These recommendations were based on expert opinion, and although three of the reviewed studies started with 100- to 400-m running increments and found participants made rehabilitative progress, the aim of these studies was not necessarily to compare a walk-run progression to another approach for increasing running distance [54, 66, 90]. Beginning with two 30- to 60-s running increments, interspersed with walking, has been recommended in recent return to competitive distance running guidelines [116]. As little as a few minutes of impact exercise will stimulate bone formation; however, bone cells become desensitised to prolonged mechanical stimulation [117]. Bone is a living tissue that can fatigue quickly so incorporating rest intervals to prevent bone fatigue is important [57, 110, 117–119]. Mechanical fatigue tests support that bone is better able to withstand mechanical loads when applied over shorter durations [110]. These points support the inclusion of a walk-run progression consisting of short-duration running periods initially, such as 30–60 s, with adequate recovery, such as 60 s, interspersed throughout.

Introducing running-related loads on alternate days, or at a reduced frequency, also has a scientific basis, as periods of relative rest enable the bone cells to regain mechanosensitivity to support further bone adaptation [108, 120]. After 24 h of rest, 98% of bone mechanosensitivity returns [120]. Muscular fatigue is thought to increase bone stress, and therefore it is important to utilise rest breaks during return to running [47, 121, 122]. It is important to repeat each level of the walk-run program several times to allow the tibia to adapt to the increasing loads. Females in particular have been shown to experience a greater increase in tibial stress when progressing from walking to running [98], as would be the case when beginning a return-to-run program, indicating that females may benefit from a slower progression initially. It may be beneficial for females to spend longer at each step of the walk-run progression or progress in smaller increments to allow longer for the tibia to adapt to the increased stress. Individualisation of this walk-run process is important and should take into account the pre-injury training status of the athlete and the severity of the injury. Further controlled trials are required to assess the effect of different walk-run progressions following a tibial BSI to make clear recommendations. It is important that the influence of the different walk-run progressions is assessed separately in males and females.

There is evidence to suggest that increased running speed can lead to significantly higher vertical tibial acceleration [123], and increases internal tibial loads [124, 125]. Rice et al. [125] found that peak posterior stress was 14% higher during level running at 3.5 m/s than 2.5 m/s. Similarly, Meardon et al. [98] found that increasing running speed by 10–20% has resulted in up to 9% increased tibial compression and tension, and up to 26% increased shear stress. The greater magnitudes of tibial acceleration and stresses at faster speeds may increase the risk of BSI. However, it should be acknowledged that those experiencing high impacts may not always develop injury.

#### Consideration 2: Progression of Load

Monitoring for the presence of pain, either during or following running, was consistently recommended to guide running progressions. Any pain suggests that the BSI site has been overloaded. If pain is present, then athletes should rest until symptoms have resolved, then resume at a lower level [2, 16, 22, 33, 41, 54, 64–66, 68, 70, 72, 76, 77, 86, 89, 91, 93]. Pain is a complex phenomenon; it is not always closely linked to musculoskeletal damage, and is mediated by numerous individual factors [126]. More precise means of monitoring patient response are needed, but at this point, pain is the only metric available to guide progression of running load following a tibial BSI.

It is recommended that running distance should be progressed before speed following a tibial BSI, which is in agreement with the six-phase *Return to Running Programme for Competitive Runners* that also recommends running distance is built to 50% of pre-injury level, before increases in speed are introduced [116]. Mechanical fatigue tests indicate that BSI risk increases more rapidly with progressions in running velocity than running distance [65, 108, 110].

While the ‘10% rule’ that guides the progression of running load in the return to running following a BSI is widely cited [34, 38, 41, 43, 45, 55, 56, 65, 77, 78, 81, 83, 86, 93], the origins of this principle are unclear. The principle appears to have originated as a progression of distance [83], but has been extrapolated across different variable domains, including time and intensity, still without supporting evidence [2, 38, 43, 77, 86]. From an injury perspective, the 10% rule appears to have originated in the space of training progression of mechanical load to prevent injury [127], and has then been translated across to the return-to-sport domain across various injuries [128, 129]. It would seem that this principle is largely based on expert opinion, as there is no empirical research providing validation. Buist et al. [130] found no difference in running injury prevalence in novice runners who followed a 10% average progression in weekly running distance, compared to those whose weekly progression was greater than this. Specific running BSI studies cite this principle as a methodology for progression of distance, as well as time and intensity, following injury, yet there is minimal evidence to support it as a guide for return to sport in general, let alone from a BSI perspective. Further to this, Nielsen et al. [131] found that tibial stress fractures were not linked to the ‘10% rule’, instead proposing they may be related to other training errors. As with many other injury- and training-related variables, runners are likely to tolerate the progression of distance, time and speed differently [108], and based on this and the evidence available, the rate of progression should be individualised and should take into account the pre-injury training status and the severity of the injury. Inadequate management of training parameters such as distance, duration, frequency and intensity, as well as the inter-relationship of these parameters, could contribute to the high recurrence rate of tibial BSIs. While the majority of the reviewed studies mentioned one or two of these parameters, the evidence across them all is lacking, and more empirical research is required to help guide progression of the return-to-running process following tibial BSIs [132–134]. Trials must assess males and females separately to provide accurate recommendations for each sex.

The rate of progression should be individualised based on risk and grade of the tibial BSI as well as the level of the athlete, which is in agreement with a meta-analysis by Hoenig et al. [28], and the return to competitive running guidelines [116]. Females may benefit from a slower progression of running due to higher tibial bone stresses across a range of running speeds, compared with males [98], and due to females often having higher-grade BSIs [24]. Where progressions are too fast, the risk of recurrence is amplified [116]. While this is generally not a factor with low-risk locations (such as posteromedial tibial BSIs) or in recreational athletes, it is an important consideration for high-risk locations (such as anterior tibial cortex BSIs) due to the increased risk of complications, or in elite athletes who are keen to return to sport as quickly as possible. Modification of the return-to-running process for athletes following high-risk tibial BSIs will require a slower overall progression and delayed integration of speedwork or resumption of the use of track spikes or racing flats. Rehabilitation should also be modified according to MRI-based injury severity if available, with a slower progression back to running for higher grade BSIs [25].

#### Consideration 3: Running Surface

With regard to surface characteristics, a quarter of studies recommended starting on a treadmill [33, 34, 68, 70, 71, 89, 93] due to the more compliant surface, and several other studies recommended avoiding hard surfaces [74, 80, 84, 85, 93]. Three studies provide supporting evidence for this recommendation, reporting that running on a treadmill resulted in a reduced peak tibial acceleration [135, 136] and lower tibial in vivo strains and strain rates [137] compared with running overground. Additionally, Milner et al. [135] reported peak tibial acceleration was lower running on the treadmill compared to grass. Conflictingly, some reviewed studies provided example return to run programmes on a running track [54, 66, 90], which is generally a hard surface. These studies were RCTs and a pilot study, and therefore the surface recommendation may simply have been to control this, as opposed to a logical or even practical solution for running.

There is conflicting evidence regarding the influence of running surface on tibial acceleration and tibial BSI risk. Harder running surfaces such as concrete have been shown to result in higher tibial acceleration [123]. Significantly lower vertical tibial acceleration has been shown during running on the woodchip trail in comparison with a synthetic running track and concrete, at least at some running velocities [123]. However, Waite et al. [138] found higher tibial acceleration on grass than concrete when running on level ground, and further studies found no difference in tibial acceleration between grass and sidewalk [135] or between dirt, gravel and paved surfaces [139]. The relationship between surface hardness and injury risk is complex as some runners compensate for different running surfaces by altering leg stiffness depending on the surface compliance [140–142]. Potthast et al. [143] concluded that surface compliance explained less than 10% of tibial acceleration variance; instead, knee joint angle and muscle pre-activation changes had greater effects on the severity of tibial acceleration. This research has been completed in healthy individuals, and it is not BSI-specific. Therefore, at this point, the influence of surfaces in the process of returning to running post-injury is conflicting and unclear. There is a lack of evidence for running on softer surfaces, which is a common presumption and recommendation; therefore, at this stage, this recommendation is unfounded.

In the initial stages post-injury, several reviewed studies have recommended it may be beneficial to avoid hills [2, 38, 43, 73]. However, once again there is conflicting evidence in the literature regarding the influence of surface incline grade. Rice et al. [125] found that running uphill at 10% and 15% inclines resulted in greater tibial stress than level running; however, Waite et al. [138] reported no difference in peak tibial acceleration between running on an incline grade compared to a level grade. In regards to running downhill, several studies found downhill surfaces to result in lower tibial stresses than level or uphill running [125, 144]. However, Waite et al. [138] found a significant increase in peak tibial acceleration on downhill surfaces compared to uphill surfaces. Further research is needed in both female and male athletes following a tibial BSI to make clear recommendations regarding the influence of different running surfaces.

#### Consideration 4: Addressing Biomechanical and Strength Factors

An important component of the return-to-running process acknowledged by almost all of the reviewed studies was the need to address biomechanical abnormalities and muscle imbalances potentially contributing to the initial injury. A recent systematic review and meta-analysis has concluded that ground reaction force variables were not different in runners with tibial BSIs compared to controls [145]. However, with regard to running gait parameters, there is some evidence to suggest that greater peak hip adduction and rearfoot eversion angles [48, 58, 146] may be associated with the development of tibial BSIs in female runners. Also, increased tibial accelerations have been shown in runners with a history of tibial BSI [59, 147, 148]. Therefore, screening for and, where appropriate, interventions aimed at addressing these variables could be beneficial during the return-to-running process. Running gait analysis and retraining were recommended by a number of reviewed studies [2, 36, 41, 43, 45, 64, 65, 67, 74, 76, 77, 79, 81, 91], and while several potentially beneficial adjustments were suggested, including reducing stride length or increasing cadence [2, 64, 65, 77] (Table 3) to reduce tibial stresses, it is beyond the scope of this review to detail all potential solutions.

Reduced lower extremity muscle size and strength have been shown to be associated with a higher risk of BSI [11, 12, 95, 102]. It is hypothesised that muscle provides a protective mechanism with respect to tibial BSIs by attenuating shock and reducing loads [108]. Additionally, resistance training has shown positive effects on increasing the strength through the shaft of a bone in females, where BSIs are more likely to occur in runners [65, 149]. If muscular activity produces dynamic mechanical signals of significant magnitudes and significant rates, it is hypothesised osteogenesis will occur [150]; therefore, resistance training should be an important component of the return-to-running process. Addressing core and proximal strength, as recommended by reviewed studies, is also important to optimise lower extremity biomechanics. Excessive hip adduction during the running gait has been identified as a predictor of tibial BSIs in female runners [146], indicating the potential need to address this in an individual with an increased adduction moment. There is inconclusive evidence regarding the effects of hip strengthening on kinematic variables, but it may improve eccentric control and could be beneficial in certain athletes [45, 151]. Cameron et al. [152] found that female and male military cadets with greater than 5° of internal knee rotation at 15%, 50% and 85% of the stance phase of a jump-landing task experienced two to four times higher stress fracture rates compared to those with neutral or external knee rotation alignment. Similarly, male and female military cadets with neutral or varus knee alignment also experienced incidence rates for stress fracture that were 43–53% lower at initial contact, 50% and 85% of stance phase during a jump-landing task compared to those with greater than 5° of knee valgus. This indicates that proximal strengthening to control these biomechanical variables may be beneficial when treating athletes with BSIs. Future research is required to assess the influence of hip and proximal strengthening on BSI incidence, particularly in female athletes.

Although only 21% of reviewed studies recommended progressing to plyometric strengthening, there is evidence from other related studies that running does not subject the body to high enough impacts to produce osteogenic effects [117]. Bilateral bone loss peaks around 12 weeks post-injury, which often coincides with a progressive return to activity [153]. There is a body of evidence from related studies that would suggest the addition of plyometric training is an area that needs attention, both practically and from a research perspective. High-impact training (defined as loads greater than four times body weight) [154] such as jumping or hopping can be highly osteogenic and energy efficient, and therefore may be beneficial for improving lower extremity bone mass during advanced stages of the return-to-running process [149, 155, 156]. Structured exercise programmes that combine high-impact loading with resistance training are effective at significantly improving bone mineral density at the lumbar spine and femoral neck in premenopausal women [157]. For the tibia specifically, zig-zag hopping, based on the high strain and strain rates that it produces, may be an optimal tibial bone-strengthening exercise [158]. It is important that jumping focuses on power as opposed to landing heavily, and only a few repetitions should be included [149, 159]. Two to four short exercise sessions per week (30 min/day or less) over at least 16 weeks are required to maintain or improve bone [155]. However, there is currently a lack of evidence suggesting whether plyometrics help enhance bone properties in runners with underlying LEA as LEA has been shown to suppress markers of bone formation in endurance athletes, in particular female athletes [156]. Therefore, investigating the addition of plyometric loading following tibial BSI is an area of future research since bone may not adapt to applied biomechanical loads if energy is not available.

### Limitations

While evaluation of the risk of bias is not mandatory for scoping reviews, 81% of studies included in this scoping review were clinical commentaries or reviews, so will inherently have a high risk of bias. Studies evaluating general lower extremity BSIs were also included in this scoping review due to the lack of studies assessing tibial BSIs. The proposed continuum from medial tibial stress syndrome (MTSS) into lower-grade bone stress reactions is still lacking evidence, and MTSS is characterised by different bone histology and clinical presentation than a BSI [160]. Therefore, for the purpose of this review, we set the line at lower-grade stress reactions (grade 1). For most classification systems, the first three grades are considered ‘stress reactions’, and when there is a visible fracture line, the injury is considered a ‘stress fracture’, and typically classified as grade 4 [22–25]. All included studies provided some guidance in terms of criteria prior to introducing running-related loads, or on the process of returning to running, following a tibial or lower extremity BSI; however, no studies in this review specifically compared return-to-running approaches. This scoping review reported criteria and guidelines for the return-to-run process based on what is recommended in published research. Other innovative and potentially useful tests and guidelines for the process of returning athletes to running following a tibial BSI may be used in practice, but are not reflected in this review.

## Conclusion and Further Recommendations

The literature has been grouped into five themes regarding the components involved in the decision on when it is appropriate to introduce running-related loads in athletes following a tibial BSI. These components include resolution of localised tibial tenderness, pain-free walking, evidence of radiological healing, assessment of lower extremity strength and pain provocation tests, and identification of contributing factors. The literature has then been grouped into four considerations involved in the process of returning an athlete to running following a tibial BSI. These considerations include beginning with a walk-run progression; individualising progression of load based on pain, risk of location, grade and level of the runner; running surface; and addressing biomechanics and strength. These components and considerations are based on level IV papers, and therefore RCTs are sorely needed in the area of returning athletes to running post tibial BSI. Deciding when an athlete is ready to return to running should be a shared decision between clinicians, coaches and athletes. Effective planning should involve addressing the athlete’s risk profile and managing risk by balancing the athlete’s interests and reinjury prevention. A multidisciplinary approach is essential to reduce the risk of recurrence. Although there is a lack of consistency or strong evidence, this review highlights the fundamental principles for returning athletes to running following a tibial BSI. The increased incidence and recurrence of BSIs in female athletes indicate there are female-specific factors that increase risk, and justify that treatment response needs to be female-specific. General sport science papers have highlighted the dearth of female research [161]; therefore, with tibial BSIs where there are obvious female-specific factors, there needs to be female-specific research. Further research, including insight from experienced practitioners, is required to develop robust guidelines for returning females to running following a tibial BSI.

## Supplementary Information

Below is the link to the electronic supplementary material.

Supplementary file1 (DOCX 16 KB)

## Data Availability

No data available.

## References

[1] Hoenig T, Ackerman KE, Beck BR, Bouxsein ML, Burr DB, Hollander K, et al. Bone stress injuries. Nat Rev Dis Primers. 2022;8(1):26. 35484131 10.1038/s41572-022-00352-y PMC13227457

[2] Warden SJ, Davis IS, Fredericson M. Management and prevention of bone stress injuries in long-distance runners. J Orthop Sports Phys Ther. 2014;44(10):749–65. 25103133 10.2519/jospt.2014.5334

[3] Warden SJ, Hoenig T, Sventeckis AM, Ackerman KE, Tenforde AS. Not all bone overuse injuries are stress fractures: it is time for updated terminology. Br J Sports Med. 2023;57(2):76–7. 36376061 10.1136/bjsports-2022-106112 PMC9812969

[4] Bennell KL, Brukner PD. Epidemiology and site specificity of stress fractures. Clin Sports Med. 1997;16(2):179–96. 9238304 10.1016/S0278-5919(05)70016-8

[5] Matheson G, Clement D, McKenzie D, Taunton J, Lloyd-Smith D, Macintyre J. Stress fractures in athletes: a study of 320 cases. Am J Sports Med. 1987;15(1):46–58. 3812860 10.1177/036354658701500107

[6] Kaeding CC, Najarian RG. Stress fractures: classification and management. Phys Sportsmed. 2010;38(3):45–54. 20959695 10.3810/psm.2010.10.1807

[7] Pegrum J, Crisp T, Padhiar N. Diagnosis and management of bone stress injuries of the lower limb in athletes. Br Med J. 2012;344: e2511. 22532009 10.1136/bmj.e2511

[8] Abbott A, Bird ML, Wild E, Brown SM, Stewart G, Mulcahey MK. Part I: epidemiology and risk factors for stress fractures in female athletes. Phys Sportsmed. 2020;48(1):17–24. 31213104 10.1080/00913847.2019.1632158

[9] Bennell KL, Malcolm SA, Thomas SA, Wark JD, Brukner PD. The incidence and distribution of stress fractures in competitive track and field athletes: a twelve-month prospective study. Am J Sports Med. 1996;24(2):211–7. 8775123 10.1177/036354659602400217

[10] Tenforde AS, Sayres LC, McCurdy ML, Collado H, Sainani KL, Fredericson M. Overuse injuries in high school runners: lifetime prevalence and prevention strategies. PM R. 2011;3(2):125–31 (**quiz 31**). 21333951 10.1016/j.pmrj.2010.09.009

[11] Bennell KL, Malcolm SA, Thomas SA, Reid SJ, Brukner PD, Ebeling PR, et al. Risk factors for stress fractures in track and field athletes: a twelve-month prospective study. Am J Sports Med. 1996;24(6):810–8. 8947404 10.1177/036354659602400617

[12] Kelsey JL, Bachrach LK, Procter-Gray E, Nieves J, Greendale GA, Sowers M, et al. Risk factors for stress fracture among young female cross-country runners. Med Sci Sports Exerc. 2007;39(9):1457–63. 17805074 10.1249/mss.0b013e318074e54b

[13] Wentz L, Liu PY, Haymes E, Ilich JZ. Females have a greater incidence of stress fractures than males in both military and athletic populations: a systemic review. Mil Med. 2011;176(4):420–30. 21539165 10.7205/MILMED-D-10-00322

[14] Tenforde AS, Sayres LC, McCurdy ML, Sainani KL, Fredericson M. Identifying sex-specific risk factors for stress fractures in adolescent runners. Med Sci Sports Exerc. 2013;45(10):1843–51. 23584402 10.1249/MSS.0b013e3182963d75

[15] Wright AA, Taylor JB, Ford KR, Siska L, Smoliga JM. Risk factors associated with lower extremity stress fractures in runners: a systematic review with meta-analysis. Br J Sports Med. 2015;49(23):1517–23. 26582192 10.1136/bjsports-2015-094828

[16] Beck B, Drysdale L. Risk factors, diagnosis and management of bone stress injuries in adolescent athletes: a narrative review. Sports (Basel). 2021;9(4):52. 33923520 10.3390/sports9040052 PMC8073721

[17] Mountjoy M, Sundgot-Borgen J, Burke L, Carter S, Constantini N, Lebrun C, et al. The IOC consensus statement: beyond the female athlete triad-relative energy deficiency in sport (RED-S). Br J Sports Med. 2014;48(7):491–7. 24620037 10.1136/bjsports-2014-093502

[18] Ackerman KE, Cano Sokoloff N, Nardo Maffazioli GDE, Clarke HM, Lee H, Misra M. Fractures in relation to menstrual status and bone parameters in young athletes. Med Sci Sports Exerc. 2015;47(8):1577–86. 25397605 10.1249/MSS.0000000000000574 PMC4430468

[19] Barrack MT, Gibbs JC, De Souza MJ, Williams NI, Nichols JF, Rauh MJ, et al. Higher incidence of bone stress injuries with increasing female athlete triad-related risk factors: a prospective multisite study of exercising girls and women. Am J Sports Med. 2014;42(4):949–58. 24567250 10.1177/0363546513520295

[20] Mountjoy M, Sundgot-Borgen J, Burke L, Ackerman KE, Blauwet C, Constantini N, et al. International Olympic Committee (IOC) Consensus Statement on relative energy deficiency in sport (RED-S): 2018 update. Int J Sport Nutr Exerc Metab. 2018;28(4):316–31. 29771168 10.1123/ijsnem.2018-0136

[21] Heikura IA, Uusitalo ALT, Stellingwerff T, Bergland D, Mero AA, Burke LM. Low energy availability is difficult to assess but outcomes have large impact on bone injury rates in elite distance athletes. Int J Sport Nutr Exerc Metab. 2018;28(4):403–11. 29252050 10.1123/ijsnem.2017-0313

[22] Arendt E, Agel J, Heikes C, Griffiths H. Stress injuries to bone in college athletes: a retrospective review of experience at a single institution. Am J Sports Med. 2003;31(6):959–68. 14623664 10.1177/03635465030310063601

[23] Fredericson M, Bergman AG, Hoffman KL, Dillingham MS. Tibial stress reaction in runners. Correlation of clinical symptoms and scintigraphy with a new magnetic resonance imaging grading system. Am J Sports Med. 1995;23(4):472–81. 7573660 10.1177/036354659502300418

[24] Nattiv A, Kennedy G, Barrack MT, Abdelkerim A, Goolsby MA, Arends JC, et al. Correlation of MRI grading of bone stress injuries with clinical risk factors and return to play: a 5-year prospective study in collegiate track and field athletes. Am J Sports Med. 2013;41(8):1930–41. 23825184 10.1177/0363546513490645 PMC4367232

[25] Hoenig T, Tenforde AS, Strahl A, Rolvien T, Hollander K. Does magnetic resonance imaging grading correlate with return to sports after bone stress injuries? A systematic review and meta-analysis. Am J Sports Med. 2022;50(3):834–44. 33720786 10.1177/0363546521993807

[26] Boden BP, Osbahr DC. High-risk stress fractures: evaluation and treatment. J Am Acad Orthop Surg. 2000;8(6):344–53. 11104398 10.5435/00124635-200011000-00002

[27] Boden BP, Osbahr DC, Jimenez C. Low-risk stress fractures. Am J Sports Med. 2001;29(1):100–11. 11206247 10.1177/03635465010290010201

[28] Hoenig T, Eissele J, Strahl A, Popp K, Stürznickel J, Ackerman K, et al. Return to sport following low-risk and high-risk bone stress injuries: a systematic review and meta-analysis. Br J Sports Med. 2023;57(7):427. 36720584 10.1136/bjsports-2022-106328

[29] Mujika I, Padilla S. Detraining: loss of training-induced physiological and performance adaptations. Part I: short term insufficient training stimulus. Sports Med. 2000;30(2):79–87. 10966148 10.2165/00007256-200030020-00002

[30] Bennell K, Crossley K, Jayarajan J, Walton E, Warden S, Kiss ZS, et al. Ground reaction forces and bone parameters in females with tibial stress fracture. Med Sci Sports Exerc. 2004;36(3):397–404. 15076780 10.1249/01.MSS.0000117116.90297.E1

[31] Ardern CL, Glasgow P, Schneiders A, Witvrouw E, Clarsen B, Cools A, et al. 2016 consensus statement on return to sport from the First World Congress in Sports Physical Therapy. Bern Br J Sports Med. 2016;50(14):853–64. 27226389 10.1136/bjsports-2016-096278

[32] Baggaley M, Vernillo G, Martinez A, Horvais N, Giandolini M, Millet GY, et al. Step length and grade effects on energy absorption and impact attenuation in running. Eur J Sport Sci. 2020;20(6):756–66. 31549912 10.1080/17461391.2019.1664639

[33] Brukner P. Exercise-related lower leg pain: bone. Med Sci Sports Exerc. 2000;32(3 Suppl):S15-s26. 10730991 10.1097/00005768-200003001-00004

[34] Carmont MR, Mei-Dan O, Bennell KL. Stress fracture management: current classification and new healing modalities. Oper Tech Sports Med. 2009;17(2):81–9. 10.1053/j.otsm.2009.05.004

[35] Chen TL, An WW, Chan ZYS, Au IPH, Zhang ZH, Cheung RTH. Immediate effects of modified landing pattern on a probabilistic tibial stress fracture model in runners. Clin Biomech. 2016;33:49–54. 10.1016/j.clinbiomech.2016.02.013 26945721

[36] Chen Y-T, Tenforde AS, Fredericson M. Update on stress fractures in female athletes: epidemiology, treatment, and prevention. Curr Rev Musculoskelet Med. 2013;6(2):173–81. 23536179 10.1007/s12178-013-9167-x PMC3702771

[37] Chisin R, Milgrom C, Giladi M, Stein M, Margulies J, Kashtan H. Clinical significance of nonfocal scintigraphic findings in suspected tibial stress fractures. Clin Orthop Relat Res. 1987;220:200–5. 10.1097/00003086-198707000-00027 3594991

[38] Couture CJ, Karlson KA. Tibial stress injuries: decisive diagnosis and treatment of “shin splints.” Phys Sportsmed. 2002;30(6):29–36. 20086529 10.3810/psm.2002.06.337

[39] Fu W, Fang Y, Liu DMS, Wang L, Ren S, Liu Y. Surface effects on in-shoe plantar pressure and tibial impact during running. J Sport Health Sci. 2015;4(4):384–90. 10.1016/j.jshs.2015.09.001

[40] Grimston SK, Engsberg JR, Kloiber R, Hanley DA. Bone mass, external loads, and stress fracture in female runners. J Appl Biomech. 1991;7(3):293–302.

[41] Harmon KG. Lower extremity stress fractures. Clin J Sport Med. 2003;13(6):358–64. 14627867 10.1097/00042752-200311000-00004

[42] Ivkovic A, Bojanic I, Pecina M. Stress fractures of the femoral shaft in athletes: a new treatment algorithm. Br J Sports Med. 2006;40(6):518. 16720887 10.1136/bjsm.2005.023655 PMC2465093

[43] Kahanov L, Eberman LE, Games KE, Wasik M. Diagnosis, treatment, and rehabilitation of stress fractures in the lower extremity in runners. Open Access J Sports Med. 2015;6:87–95. 25848327 10.2147/OAJSM.S39512 PMC4384749

[44] Kiuru MJ, Pihlajamäki H, Ahovuo J. Bone stress injuries. Acta Radiol. 2004;45(3). 10.1080/02841850410004724 15239429

[45] Liem BC, Truswell HJ, Harrast MA. Rehabilitation and return to running after lower limb stress fractures. Curr Sports Med Rep. 2013;12(3):200–7. 23669091 10.1249/JSR.0b013e3182913cbe

[46] McMahon TA, Greene PR. The influence of track compliance on running. J Biomech. 1979;12(12):893–904. 528547 10.1016/0021-9290(79)90057-5

[47] Milgrom C, Radeva-Petrova DR, Finestone A, Nyska M, Mendelson S, Benjuya N, et al. The effect of muscle fatigue on in vivo tibial strains. J Biomech. 2007;40(4):845–50. 16682046 10.1016/j.jbiomech.2006.03.006

[48] Milner CE, Hamill J, Davis IS. Distinct hip and rearfoot kinematics in female runners with a history of tibial stress fracture. J Orthop Sports Phys Ther. 2010;40(2):59–66. 20118528 10.2519/jospt.2010.3024

[49] Moreira CA, Bilezikian JP. Stress fractures: concepts and therapeutics. J Clin Endocrinol Metab. 2017;102(2):525–34. 27732325 10.1210/jc.2016-2720

[50] Peters MDJ, Marnie C, Tricco AC, Pollock D, Munn Z, Alexander L, et al. Updated methodological guidance for the conduct of scoping reviews. JBI Evid Synth. 2020;18(10):2119–26. 33038124 10.11124/JBIES-20-00167

[51] Sheerin KR, Besier TF, Reid D. The influence of running velocity on resultant tibial acceleration in runners. Sports Biomech. 2020;19(6):750–60. 30537920 10.1080/14763141.2018.1546890

[52] Song SH, Koo JH. Bone stress injuries in runners: a review for raising interest in stress fractures in Korea. J Korean Med Sci. 2020;35(8): e38. 32103643 10.3346/jkms.2020.35.e38 PMC7049623

[53] Sterling JC, Edelstein DW, Calvo RD, Webb R 2nd. Stress fractures in the athlete. Diagnosis and management. Sports Med. 1992;14(5):336–46. 1439400 10.2165/00007256-199214050-00005

[54] Swenson EJ Jr, DeHaven KE, Sebastianelli WJ, Hanks G, Kalenak A, Lynch JM. The effect of a pneumatic leg brace on return to play in athletes with tibial stress fractures. Am J Sports Med. 1997;25(3):322–8. 9167811 10.1177/036354659702500309

[55] Taube RR, Wadsworth TL, Johnson RJ. Managing tibial stress fractures. Phys Sportsmed. 1993;21(4):123–30. 27447774 10.1080/00913847.1993.11710368

[56] Toresdahl BG, Nguyen J, Goolsby MA, Drakos MC, Lyman S. High number of daily steps recorded by runners recovering from bone stress injuries. HSS J. 2020;16(Suppl 2):408–11. 33380974 10.1007/s11420-020-09787-z PMC7749902

[57] Turner CH, Robling AG. Exercises for improving bone strength. Br J Sports Med. 2005;39(4):188–9. 15793082 10.1136/bjsm.2004.016923 PMC1725178

[58] Willwacher S, Kurz M, Robbin J, Thelen M, Hamill J, Kelly L, et al. Running-related biomechanical risk factors for overuse injuries in distance runners: a systematic review considering injury specificity and the potentials for future research. Sports Med. 2022;52(8):1863–77. 35247202 10.1007/s40279-022-01666-3 PMC9325808

[59] Zadpoor AA, Nikooyan AA. The relationship between lower-extremity stress fractures and the ground reaction force: a systematic review. Clin Biomech. 2011;26(1):23–8. 10.1016/j.clinbiomech.2010.08.005 20846765

[60] Zahger D, Abramovitz A, Zelikovsky L, Israel O, Israel P. Stress fractures in female soldiers: an epidemiological investigation of an outbreak. Mil Med. 1988;153(9):448–50. 2847081 10.1093/milmed/153.9.448

[61] Arksey H, O’Malley L. Scoping studies: towards a methodological framework. Int J Soc Res Methodol. 2005;8(1):19–32. 10.1080/1364557032000119616

[62] Peters M, Godfrey C, McInerney P, Soares C, Khalil H, Parker D. Methodology for JBI scoping reviews. In: Aromataris E, editor. The Joanna Briggs Institute Reviewers’ manual 2015. Adelaide: The Joanna Briggs Institute; 2015. p. 1–24.

[63] Braun V, Clarke V. Using thematic analysis in psychology. Qual Res Psychol. 2006;3(2):77–101. 10.1191/1478088706qp063oa

[64] Bolthouse E, Hunt A, Mandrachia K, Monarski L, Lee K. Return to running after a tibial stress fracture: a suggested protocol. Orthop Phys Ther Pract. 2015;27(1):37–45.

[65] Warden SJ, Edwards WB, Willy RW. Optimal load for managing low-risk tibial and metatarsal bone stress injuries in runners: the science behind the clinical reasoning. J Orthop Sports Phys Ther. 2021;51(7):322–30. 33962529 10.2519/jospt.2021.9982

[66] Allen CS, Flynn TW, Kardouni JR, Hemphill MH, Schneider CA, Pritchard AE, et al. The use of a pneumatic leg brace in soldiers with tibial stress fractures—a randomized clinical trial. Mil Med. 2004;169(11):880–4. 15605935 10.7205/MILMED.169.11.880

[67] Beck BR. Tibial stress injuries. An aetiological review for the purposes of guiding management. Sports Med. 1998;26(4):265–79. 9820925 10.2165/00007256-199826040-00005

[68] Brukner P, Bennell K. Stress fractures in female athletes: diagnosis, management and rehabilitation. Sports Med. 1997;24(6):419–29. 9421865 10.2165/00007256-199724060-00006

[69] Brukner P, Bradshaw C, Bennell K. Managing common stress fractures: let risk level guide treatment. Phys Sportsmed. 1998;26(8):39–47. 20086841 10.3810/psm.1998.08.1104

[70] Brukner PD, Bennell KL. Review on stress fractures. Crit Rev Phys Rehabil Med. 2017;29(1–4):143–87.

[71] Corrarino JE. Stress fractures in runners. Nurse Pract. 2012;37(6):18–28. 22546779 10.1097/01.NPR.0000414593.94152.e8

[72] Flinn SD. Changes in stress fracture distribution and current treatment. Curr Sports Med Rep. 2002;1(5):272–7. 12831689 10.1249/00149619-200210000-00004

[73] Fredericson M, Jennings F, Beaulieu C, Matheson GO. Stress fractures in athletes. Top Magn Reson Imaging. 2006;17(5):309–25. 17414993 10.1097/RMR.0b013e3180421c8c

[74] Harrast MA, Colonno D. Stress fractures in runners. Clin J Sport Med. 2010;29(3):399–416. 10.1016/j.csm.2010.03.001 20610029

[75] Heaslet MW, Kanda-Mehtani SL. Return-to-activity levels in 96 athletes with stress fractures of the foot, ankle, and leg: a retrospective analysis. J Am Podiatr Med Assoc. 2007;97(1):81–4. 17218629 10.7547/0970081

[76] Hoch AZ, Pepper M, Akuthota V. Stress fractures and knee injuries in runners. Phys Med Rehabil Clin N Am. 2005;16(3):749–77. 16005402 10.1016/j.pmr.2005.02.008

[77] Jasty N, Dyrek P, Kaur J, Ackerman KE, Kraus E, Heyworth B. Evidence-based treatment and outcomes of tibial bone stress injuries. J Pediatr Soc N Am. 2021;3(4):372.

[78] McCormick F, Nwachukwu BU, Provencher MT. Stress fractures in runners. Clin Sports Med. 2012;31(2):291–306. 22341018 10.1016/j.csm.2011.09.012

[79] Miller TL, Best TM. Taking a holistic approach to managing difficult stress fractures. J Orthop Surg Res. 2016;11(1):98. 27608681 10.1186/s13018-016-0431-9 PMC5016928

[80] Nelson BJ, Arciero RA. Stress fractures in the female athlete. Sports Med Arthrosc Rev. 2002;10(1):83–90. 10.1097/00132585-200210010-00012

[81] Nicola TL, El Shami A. Rehabilitation of running injuries. Clin Sports Med. 2012;31(2):351–72. 22341022 10.1016/j.csm.2011.10.002

[82] Patel DR. Stress fractures: diagnosis and management in the primary care setting. Pediatr Clin N Am. 2010;57(3):819–27. 10.1016/j.pcl.2010.03.004 20538158

[83] Paty JG Jr. Diagnosis and treatment of musculoskeletal running injuries. Semin Arthritis Rheum. 1988;18(1):48–60. 3055303 10.1016/0049-0172(88)90034-0

[84] Reeder MT, Dick BH, Atkins JK, Pribis AB, Martinez JM. Stress fractures. Current concepts of diagnosis and treatment. Sports Med. 1996;22(3):198–212. 8883216 10.2165/00007256-199622030-00006

[85] Romani WA, Gieck JH, Perrin DH, Saliba EN, Kahler DM. Mechanisms and management of stress fractures in physically active persons. J Athl Train. 2002;37(3):306–14. 16558676 PMC164361

[86] Valente C, Reis E, Filho D. Stress fracture in non-athletes: a systematic review. MedNEXT J Med Health Sci. 2021;2:32–41.

[87] van der Velde GM, Hsu WS. Posterior tibial stress fracture: a report of three cases. J Manipulative Physiol Ther. 1999;22(5):341–6. 10395437 10.1016/S0161-4754(99)70067-9

[88] Tsakotos GA, Tokis AV, Paganias CG. Tension band plating of an anterior tibial stress fracture nonunion in an elite athlete, initially treated with intramedullary nailing: a case report. J Med Case Rep. 2018;12(1):183. 29954458 10.1186/s13256-018-1718-8 PMC6025831

[89] Bennell K, Brukner P. Preventing and managing stress fractures in athletes. Phys Ther Sport. 2005;6(4):171–80. 10.1016/j.ptsp.2005.07.002

[90] Brown WJ, Lewis PC, Neugebauer-Sperlein J, Zarow GJ, Rivas E. A novel stress fracture rehabilitation program: a pilot study. Mil Med. 2021;186:820–7. 33499545 10.1093/milmed/usaa449

[91] Arendt EA, Griffiths HJ. The use of MR imaging in the assessment and clinical management of stress reactions of bone in high-performance athletes. Clin J Sport Med. 1997;16(2):291–306. 10.1016/S0278-5919(05)70023-5 9238311

[92] O’Toole ML. Prevention and treatment of injuries to runners. Med Sci Sports Exerc. 1992;24(9):S360–3. 1406210 10.1249/00005768-199209001-00010

[93] Raasch WG, Hergan DJ. Treatment of stress fractures: the fundamentals. Clin J Sport Med. 2006;25(1):29–36, vii. 10.1016/j.csm.2005.08.013 16324971

[94] Head PL, Ploor R. Treatment of a bone stress injury with low-intensity pulsed ultrasound (LIPUS). Int J Athl Ther Train. 2012;17:35–9. 10.1123/ijatt.17.6.35

[95] Beck BR, Rudolph K, Matheson GO, Bergman AG, Norling TL. Risk factors for tibial stress injuries: a case-control study. Clin J Sport Med. 2015;25(3):230. 24977954 10.1097/JSM.0000000000000126

[96] Anderson MW, Ugalde V, Batt M, Greenspan A. longitudinal stress fracture of the tibia: MR demonstration. J Comput Assist Tomogr. 1996;20(5):836–8. 8797928 10.1097/00004728-199609000-00034

[97] Knobloch K, Schreibmueller L, Jagodzinski M, Zeichen J, Krettek C. Rapid rehabilitation programme following sacral stress fracture in a long-distance running female athlete. Arch Orthop Trauma Surg. 2007;127(9):809–13. 16906424 10.1007/s00402-006-0201-y

[98] Meardon SA, Derrick TR, Willson JD, Baggaley M, Steinbaker CR, Marshall M, et al. Peak and per-step tibial bone stress during walking and running in female and male recreational runners. Am J Sports Med. 2021;49(8):2227–37. 34077287 10.1177/03635465211014854

[99] Kaeding CC, Yu JR, Wright R, Amendola A, Spindler KP. Management and return to play of stress fractures. Clin J Sport Med. 2005;15(6):442–7. 16278549 10.1097/01.jsm.0000188207.62608.35

[100] Niemeyer P, Weinberg A, Schmitt H, Kreuz PC, Ewerbeck V, Kasten P. Stress fractures in adolescent competitive athletes with open physis. Knee Surg Sports Traumatol Arthrosc. 2006;14(8):771–7. 16328465 10.1007/s00167-005-0003-8

[101] Wright AA, Hegedus EJ, Lenchik L, Kuhn KJ, Santiago L, Smoliga JM. Diagnostic accuracy of various imaging modalities for suspected lower extremity stress fractures: a systematic review with evidence-based recommendations for clinical practice. Am J Sports Med. 2016;44(1):255–63. 25805712 10.1177/0363546515574066

[102] Hoffman JR, Chapnik L, Shamis A, Givon U, Davidson B. The effect of leg strength on the incidence of lower extremity overuse injuries during military training. Mil Med. 1999;164(2):153–6. 10050576 10.1093/milmed/164.2.153

[103] Warden SJ, Burr DB, Brukner PD. Stress fractures: pathophysiology, epidemiology, and risk factors. Curr Osteoporos Rep. 2006;4(3):103–9. 16907999 10.1007/s11914-996-0029-y

[104] Bradley LN, Blakey SC. Single-leg hop test in the evaluation of stress injuries. Athl Train Sports Health Care. 2014;6(5):201–2. 10.3928/19425864-20140916-11

[105] Nussbaum ED, Gatt CJ, Bjornarra J, Yang C. Evaluating the clinical tests for adolescent tibial bone stress injuries. Sports Health. 2021;13(5):502–10. 33576312 10.1177/1941738120988691 PMC8404772

[106] Kahanov L, Eberman L, Alvey T, True J, Yeargin B. Sacral stress fracture in a distance runner. J Osteopath Med. 2011;111(10):585–91. 22065300

[107] Bertelsen M, Hulme A, Petersen J, Brund RK, Sørensen H, Finch C, et al. A framework for the etiology of running-related injuries. Scand J Med Sci Sports. 2017;27(11):1170–80. 28329441 10.1111/sms.12883

[108] Warden SJ, Edwards WB, Willy RW. Preventing bone stress injuries in runners with optimal workload. Curr Osteoporos Rep. 2021;19(3):298–307. 33635519 10.1007/s11914-021-00666-y PMC8316280

[109] Hreljac A. Etiology, prevention, and early intervention of overuse injuries in runners: a biomechanical perspective. Phys Med Rehabil Clin. 2005;16(3):651–67. 10.1016/j.pmr.2005.02.002 16005398

[110] Edwards WB. Modeling overuse injuries in sport as a mechanical fatigue phenomenon. Exerc Sport Sci Rev. 2018;46(4):224–31. 30001271 10.1249/JES.0000000000000163

[111] Melin A, Tornberg AB, Skouby S, Faber J, Ritz C, Sjödin A, et al. The LEAF questionnaire: a screening tool for the identification of female athletes at risk for the female athlete triad. Br J Sports Med. 2014;48(7):540–5. 24563388 10.1136/bjsports-2013-093240

[112] De Souza MJ, Nattiv A, Joy E, Misra M, Williams NI, Mallinson RJ, et al. 2014 Female athlete triad coalition consensus statement on treatment and return to play of the female athlete triad: 1st international conference held in San Francisco, California, May 2012 and 2nd international conference held in Indianapolis, Indiana, May 2013. Br J Sports Med. 2014;48(4):289. 24463911 10.1136/bjsports-2013-093218

[113] Mountjoy M, Sundgot-Borgen J, Burke L, Carter S, Constantini N, Lebrun C, et al. The IOC relative energy deficiency in sport clinical assessment tool (RED-S CAT). Br J Sports Med. 2015;49:1354. 26764434 10.1136/bjsports-2015-094873

[114] Tenforde AS, Carlson JL, Chang A, Sainani KL, Shultz R, Kim JH, et al. Association of the female athlete triad risk assessment stratification to the development of bone stress injuries in collegiate athletes. Am J Sports Med. 2017;45(2):302–10. 28038316 10.1177/0363546516676262

[115] Koltun KJ, Strock NC, Southmayd EA, Oneglia AP, Williams NI, De Souza MJ. Comparison of female athlete triad coalition and RED-S risk assessment tools. J Sports Sci. 2019;37(21):2433–42. 31296115 10.1080/02640414.2019.1640551

[116] Hegedus EJ, Ickes L, Jakobs F, Ford KR, Smoliga JM. Comprehensive return to competitive distance running: a clinical commentary. Sports Med. 2021;51(12):2507–23. 34478108 10.1007/s40279-021-01547-1

[117] Boudenot A, Achiou Z, Portier H. Does running strengthen bone? Appl Physiol Nutr Metab. 2015;40(12):1309–12. 26562001 10.1139/apnm-2015-0265

[118] Burr DB, Robling AG, Turner CH. Effects of biomechanical stress on bones in animals. Bone. 2002;30(5):781–6. 11996920 10.1016/S8756-3282(02)00707-X

[119] Turner CH, Robling AG. Designing exercise regimens to increase bone strength. Exerc Sport Sci Rev. 2003;31(1):45–50. 12562170 10.1097/00003677-200301000-00009

[120] Robling AG, Burr DB, Turner CH. Recovery periods restore mechanosensitivity to dynamically loaded bone. J Exp Biol. 2001;204(Pt 19):3389–99. 11606612 10.1242/jeb.204.19.3389

[121] Mizrahi J, Verbitsky O, Isakov E. Fatigue-related loading imbalance on the shank in running: a possible factor in stress fractures. Ann Biomed Eng. 2000;28(4):463–9. 10870903 10.1114/1.284

[122] Yoshikawa T, Mori S, Santiesteban AJ, Sun TC, Hafstad E, Chen J, et al. The effects of muscle fatigue on bone strain. J Exp Biol. 1994;188(1):217–33. 7964380 10.1242/jeb.188.1.217

[123] Boey H, Aeles J, Schütte K, Vanwanseele B. The effect of three surface conditions, speed and running experience on vertical acceleration of the tibia during running. Sports Biomech. 2017;16(2):166–76. 27595311 10.1080/14763141.2016.1212918

[124] Keast M, Bonacci J, Fox A. Acute effects of gait interventions on tibial loads during running: a systematic review and meta-analysis. Sports Med. 2022;52(10):2483–509. 35708887 10.1007/s40279-022-01703-1 PMC9474464

[125] Rice H, Kurz M, Mai P, Robertz L, Bill K, Derrick TR, et al. Speed and surface steepness affect internal tibial loading during running. J Sport Health Sci. 2023;13:118–24. 36931595 10.1016/j.jshs.2023.03.004 PMC10818105

[126] Marchand S. Mechanisms challenges of the pain phenomenon. Front Pain Res. 2020;1: 574370. 10.3389/fpain.2020.574370 PMC8915747 35295689

[127] American College of Sports Medicine. Current comment from the American College of Sports Medicine. August 1993—the prevention of sport injuries of children and adolescents. Med Sci Sports Exerc. 1993;25(8 Suppl):1–7. 8371662

[128] Adrian E-E, Jose C, Antonio IC-V. Load progression criteria in exercise programmes in lower limb tendinopathy: a systematic review. BMJ Open. 2020;10(11):e041433. 10.1136/bmjopen-2020-041433 PMC7678382 33444210

[129] Kraeutler MJ, Anderson J, Chahla J, Mitchell JJ, Thompson-Etzel R, Mei-Dan O, et al. Return to running after arthroscopic hip surgery: literature review and proposal of a physical therapy protocol. J Hip Preserv Surg. 2017;4(2):121–30. 28630733 10.1093/jhps/hnx012 PMC5467407

[130] Buist I, Bredeweg SW, van Mechelen W, Lemmink KA, Pepping GJ, Diercks RL. No effect of a graded training program on the number of running-related injuries in novice runners: a randomized controlled trial. Am J Sports Med. 2008;36(1):33–9. 17940147 10.1177/0363546507307505

[131] Nielsen R, Parner ET, Nohr EA, Sørensen H, Lind M, Rasmussen S. Excessive progression in weekly running distance and risk of running-related injuries: an association which varies according to type of injury. J Orthop Sports Phys Ther. 2014;44(10):739–47. 25155475 10.2519/jospt.2014.5164

[132] Nielsen RO, Buist I, Sørensen H, Lind M, Rasmussen S. Training errors and running related injuries: a systematic review. Int J Sports Phys Ther. 2012;7(1):58. 22389869 PMC3290924

[133] van Gent RN, Siem D, van Middelkoop M, van Os AG, Bierma-Zeinstra SM, Koes BW. Incidence and determinants of lower extremity running injuries in long distance runners: a systematic review. Br J Sports Med. 2007;41(8):469–80. 17473005 10.1136/bjsm.2006.033548 PMC2465455

[134] Fredette A, Roy JS, Perreault K, Dupuis F, Napier C, Esculier JF. The association between running injuries and training parameters: a systematic review. J Athl Train. 2022;57(7):650–71. 34478518 10.4085/1062-6050-0195.21 PMC9528699

[135] Milner CE, Hawkins JL, Aubol KG. Tibial acceleration during running is higher in field testing than indoor testing. Med Sci Sports Exerc. 2020;52(6):1361–6. 31913243 10.1249/MSS.0000000000002261

[136] Montgomery G, Abt G, Dobson C, Smith T, Ditroilo M. Tibial impacts and muscle activation during walking, jogging and running when performed overground, and on motorised and non-motorised treadmills. Gait Posture. 2016;49:120–6. 27400020 10.1016/j.gaitpost.2016.06.037

[137] Milgrom C, Finestone A, Segev S, Olin C, Arndt T, Ekenman I. Are overground or treadmill runners more likely to sustain tibial stress fracture? Br J Sports Med. 2003;37(2):160–3. 12663360 10.1136/bjsm.37.2.160 PMC1724607

[138] Waite N, Goetschius J, Lauver JD. Effect of grade and surface type on peak tibial acceleration in trained distance runners. J Appl Biomech. 2021;37(1):2–5. 33022655 10.1123/jab.2020-0096

[139] Garcia MC, Gust G, Bazett-Jones DM. Tibial acceleration and shock attenuation while running over different surfaces in a trail environment. J Sci Med Sport. 2021;24(11):1161–5. 33766445 10.1016/j.jsams.2021.03.006

[140] Dixon SJ, Collop AC, Batt ME. Surface effects on ground reaction forces and lower extremity kinematics in running. Med Sci Sports Exerc. 2000;32(11):1919–26. 11079523 10.1097/00005768-200011000-00016

[141] Ferris DP, Liang K, Farley CT. Runners adjust leg stiffness for their first step on a new running surface. J Biomech. 1999;32(8):787–94. 10433420 10.1016/S0021-9290(99)00078-0

[142] Ferris DP, Louie M, Farley CT. Running in the real world: adjusting leg stiffness for different surfaces. Proc R Soc Lond B Biol Sci. 1998;265(1400):989–94. 10.1098/rspb.1998.0388 PMC1689165 9675909

[143] Potthast W, Brüggemann GP, Lundberg A, Arndt A. The influences of impact interface, muscle activity, and knee angle on impact forces and tibial and femoral accelerations occurring after external impacts. J Appl Biomech. 2010;26(1):1–9. 20147752 10.1123/jab.26.1.1

[144] Baggaley M, Derrick TR, Vernillo G, Millet GY, Edwards WB. Internal tibial forces and moments during graded running. J Biomech Eng. 2022;144(1):011009. 34318310 10.1115/1.4051924

[145] Milner CE, Foch E, Gonzales JM, Petersen D. Biomechanics associated with tibial stress fracture in runners: a systematic review and meta-analysis. J Sport Health Sci. 2023;12(3):333–42. 36481573 10.1016/j.jshs.2022.12.002 PMC10199137

[146] Pohl MB, Mullineaux DR, Milner CE, Hamill J, Davis IS. Biomechanical predictors of retrospective tibial stress fractures in runners. J Biomech. 2008;41(6):1160–5. 18377913 10.1016/j.jbiomech.2008.02.001

[147] Milner CE, Ferber R, Pollard CD, Hamill J, Davis IS. Biomechanical factors associated with tibial stress fracture in female runners. Med Sci Sports Exerc. 2006;38(2):323. 16531902 10.1249/01.mss.0000183477.75808.92

[148] Van der Worp H, Vrielink JW, Bredeweg SW. Do runners who suffer injuries have higher vertical ground reaction forces than those who remain injury-free? A systematic review and meta-analysis. Br J Sports Med. 2016;50(8):450–7. 26729857 10.1136/bjsports-2015-094924

[149] Lambert C, Beck BR, Harding AT, Watson SL, Weeks BK. Regional changes in indices of bone strength of upper and lower limbs in response to high-intensity impact loading or high-intensity resistance training. Bone. 2020;132: 115192. 31846824 10.1016/j.bone.2019.115192

[150] Robling AG. Is bone’s response to mechanical signals dominated by muscle forces? Med Sci Sports Exerc. 2009;41(11):2044–9. 19812512 10.1249/MSS.0b013e3181a8c702 PMC3412134

[151] Willy RW, Davis IS. The effect of a hip-strengthening program on mechanics during running and during a single-leg squat. J Orthop Sports Phys Ther. 2011;41(9):625–32. 21765220 10.2519/jospt.2011.3470

[152] Cameron KL, Peck KY, Owens BD, Svoboda SJ, Padua DA, DiStefano LJ, et al. Biomechanical risk factors for lower extremity stress fracture. Orthop J Sports Med. 2013;1(4_suppl):2325967113S00019. 10.1177/2325967113S00019

[153] Popp KL, Ackerman KE, Rudolph SE, Johannesdottir F, Hughes JM, Tenforde AS, et al. Changes in volumetric bone mineral density over 12 months after a tibial bone stress injury diagnosis: implications for return to sports and military duty. Am J Sports Med. 2021;49(1):226–35. 33259223 10.1177/0363546520971782

[154] Witzke KA, Snow CM. Effects of plyometric jump training on bone mass in adolescent girls. Med Sci Sports Exerc. 2000;32(6):1051–7. 10862529 10.1097/00005768-200006000-00003

[155] Troy KL, Mancuso ME, Butler TA, Johnson JE. Exercise early and often: effects of physical activity and exercise on women’s bone health. Int J Environ Res Public Health. 2018;15(5):878. 29710770 10.3390/ijerph15050878 PMC5981917

[156] Hutson MJ, O’Donnell E, Brooke-Wavell K, Sale C, Blagrove RC. Effects of low energy availability on bone health in endurance athletes and high-impact exercise as a potential countermeasure: a narrative review. Sports Med. 2021;51(3):391–403. 33346900 10.1007/s40279-020-01396-4 PMC7900047

[157] Martyn-St James M, Carroll S. Effects of different impact exercise modalities on bone mineral density in premenopausal women: a meta-analysis. J Bone Miner Metab. 2010;28(3):251–67. 20013013 10.1007/s00774-009-0139-6

[158] Milgrom C, Miligram M, Simkin A, Burr D, Ekenman I, Finestone A. A home exercise program for tibial bone strengthening based on in vivo strain measurements. Am J Phys Med Rehabil. 2001;80(6):433–8. 11399004 10.1097/00002060-200106000-00009

[159] Vlachopoulos D, Barker AR, Ubago-Guisado E, Williams CA, Gracia-Marco L. The effect of a high-impact jumping intervention on bone mass, bone stiffness and fitness parameters in adolescent athletes. Arch Osteoporos. 2018;13(1):1–12. 10.1007/s11657-018-0543-4 PMC6244891 30446875

[160] Aoki Y, Yasuda K, Tohyama H, Ito H, Minami A. Magnetic resonance imaging in stress fractures and shin splints. Clin Orthop Relat Res. 2004;421:260–7. 10.1097/01.blo.0000126333.13806.87 15123957

[161] Emmonds S, Heyward O, Jones B. The challenge of applying and undertaking research in female sport. Sports Med Open. 2019;5(1):51. 31832880 10.1186/s40798-019-0224-x PMC6908527

