# Supplementary material for: Criteria and Guidelines for Returning to Running Following a Tibial Bone Stress Injury: A Scoping Review
Source: Sports Med. 2024 Aug 14;54(9):2247–65. doi: 10.1007/s40279-024-02051-y (PMC11393297; doi:10.1007/s40279-024-02051-y)
Supplement: Supplementary file 1 — Supplementary file1 (DOCX 16 KB) [file 40279_2024_2051_MOESM1_ESM.docx]

Searches completed July 2021

**Database 1:**

**EBSCO Health (MEDLINE, SPORTDiscus, CINAHL)**

"Bon* Stress Injur*" OR "Stress fracture*" OR "Stress reaction*"

AND
"lower extremit*" OR "lower limb*" OR leg* OR knee OR tibia*

AND
( (return*) n3 (sport* OR play* OR training OR activit*) ) OR run*

1074 results (CINAHL: 240, Medline 464, SPORTDiscus: 370)

767 results (after duplicates removed)

**Database 2:**

**Scopus Search:**

( TITLE-ABS-KEY ( "Bon* Stress Injur*"  OR  "Stress fracture*"  OR  "Stress reaction*" ) )

AND

( TITLE-ABS-KEY ( "lower extremit*"  OR  "lower limb*"  OR  leg*  OR  knee  OR  tibia* ) )

AND

( ( TITLE-ABS-KEY ( return*  W/3  ( sport*  OR  play*  OR  training  OR  activit* ) )  OR  TITLE-ABS-KEY ( run* ) ) )

657 results

**Database 3:**

**AMED Search Strategy:**
*Search 1:*

("Bon* Stress injur*" or "stress fracture*" or "stress reaction*").mp. [mp=abstract, heading words, title]

AND

("lower extremit*" or "lower limb*" or leg* or knee or tibia).mp. [mp=abstract, heading words, title]

AND

(return* adj3 (sport* or play or training or activit*)).mp. [mp=abstract, heading words, title]

12 articles

*Search 2:*

("Bon* Stress injur*" or "stress fracture*" or "stress reaction*").mp. [mp=abstract, heading words, title]

AND

("lower extremit*" or "lower limb*" or leg* or knee or tibia).mp. [mp=abstract, heading words, title]

AND

Run*

53 articles

63 (once the two searches were combined and duplicates removed)

Total record identified: 1794

All references: 1487 (when duplicates removed from EBSCO)

Duplicates: 824

Records after duplicates removed: 1040

*Records excluded (n=931)*

Records after titles and abstracts screened: 109 articles

*Full text studies excluded (n=63)*

*No mention of return to running (n=57)*

*Not focused on bone stress injury management (n=2)*

*Full text unavailable (n=4)*

Total studies meeting eligibility criteria: 46 studies

Google Scholar forward citation tracking: 2 extra studies added

Hand searching reference lists: 2 extra studies added

Total studies: 50 studies.
